# Cervical cancer patients' vaginal microbiome and metabolites: research progress and clinical applications

**DOI:** 10.3389/fmicb.2026.1891111

**Published:** 2026-08-14

**Authors:** Qian Huang, Li Yi Zhang, Hui Lin Shu, Na Chen, Rong Hua Li, Feng Xi Jiang, Guang Ming Li, Mei Pu, Qian Zhang, Yu Lian Gao, Yan Peng Chu

**Affiliations:** 1 Department of Immunology, School of Basic Medical Sciences, Ningxia Medical University, Yinchuan, Ningxia, China; 2 Dazhou Vocational and Technical College, Dahzou, Sichuan, China; 3 Department of Obstetrics and Gynecology, Dazhou Central Hospital, Dahzou, Sichuan, China; 4 Department of Pediatrics, Dazhou Hospital of Integrated Chinese and Western Medicine, Dahzou, Sichuan, China; 5 School of Clinical Medicine, Southwest Medical University, Luzhou, Sichuan, China; 6 Department of Cardiology, Dazhou Central Hospital, Dazhou, Sichuan, China; 7 Medical College of Sichuan University of Arts and Sciences, Dazhou, Sichuan, China

**Keywords:** biomarkers, cervical cancer, *Lactobacillus*, metabolites, pathogenesis, probiotics, vaginal microbiota

## Abstract

As the fourth most common malignancy among women globally, cervical cancer is intimately linked to persistent high-risk human papillomavirus (HPV) infection; however, HPV infection is not the sole pathogenic factor. Recent studies have revealed that the composition, dynamics, and metabolic products of the vaginal microbiota play pivotal roles in the occurrence, progression, diagnosis, and treatment of cervical cancer. This article offers a comprehensive narrative review of foundational research on the vaginal microbiome in cervical cancer patients, correlations with metabolites, epidemiological characteristics, diagnostic applications, therapeutic strategies, and ongoing controversies and challenges. Basic research demonstrates that increased vaginal microbial diversity and reduced *Lactobacillus* abundance are associated with cervical cancer progression, wherein pathogenic bacteria such as *Fusobacterium* spp. and *Sneathia* spp. have been associated with tumorigenesis through immunosuppression and inflammatory mechanisms. Metabolites like 3-hydroxybutyrate and sphingolipids show potential as diagnostic biomarkers and therapeutic targets. Epidemiological studies reveal associations between variations in the vaginal microbiota across different populations (e.g., African, Latina) and cervical cancer risk. In diagnostics, detection methods based on the vaginal microbiome can enhance early screening efficiency, with novel sequencing technologies combined with artificial intelligence demonstrating promising application prospects. Therapeutically, interventions such as probiotics and microbiota modulation may improve patient outcomes. Nonetheless, current research faces challenges, including an unclear causal relationship between the microbiota and HPV infection and a lack of standardization in detection techniques. Future endeavors necessitate multi-omics integration, large-scale longitudinal studies, and precise intervention strategies to facilitate the clinical translation of vaginal microbiome research into cervical cancer prevention and treatment.

## 1 Introduction

Cervical cancer remains a major global health burden, with an estimated 600,000 new cases and 340,000 deaths annually (Brooke et al., 2024), Persistent infection with high-risk HPV types, particularly HPV16 and HPV18, is the necessary but not sufficient cause (Kao et al., 2026); most HPV infections resolve spontaneously, and only a minority progress to high-grade intraepithelial neoplasia and invasive carcinoma. This has led to increasing interest in host and environmental co-factors (Molina et al., 2024), among which the vaginal microbial ecosystem has emerged as a key player (Sharifian et al., 2023).

The vaginal microbiota in healthy reproductive-age women is typically dominated by *Lactobacillus* species (*L. crispatus, L. iners, L. gasseri, L. jensenii*) (Bhattacharya et al., 2023), which produce lactic acid and maintain a low pH (< 4.5), creating a barrier against pathogens (Sharifian et al., 2023). However, when this homeostasis is disrupted—often termed dysbiosis or bacterial vaginosis (BV)—a polymicrobial community with high diversity and reduced lactobacilli becomes established (Moghaddam et al., 2026).

Over the past decade, numerous cross-sectional and longitudinal studies have reported associations between such dysbiotic states and HPV infection, cervical intraepithelial neoplasia (CIN), and cervical cancer (Zhang et al., 2024). Concurrently, advances in metabolomics have revealed that microbial metabolites (e.g., short-chain fatty acids, tryptophan catabolites, lipids) can influence local immune responses and epithelial cell behavior (Xu et al., 2022), adding a functional layer to the composition-based picture.

Despite the proliferation of data, the field faces several challenges: most studies are associative, definitions of “healthy” microbiota vary, and the causal direction between dysbiosis and HPV-induced carcinogenesis remains uncertain (Moghaddam et al., 2026). Moreover, translation of microbiome findings into clinical practice—as biomarkers, screening tools, or therapeutic targets—is still in its infancy (Gomes et al., 2026).

Beyond cervical cancer, microbiota involvement in other gynecological malignancies has attracted increasing research attention (Liao et al., 2026). Gynecological malignancies (cervical, endometrial, and ovarian cancers) have multifactorial etiologies driven by genetic, environmental, hormonal, and infectious factors (Wierzbińska et al., 2025). Cumulative evidence identifies microbial dysbiosis as a common pathogenic contributor to these tumors (Alhamlan et al., 2024). In healthy females, the reproductive tract is dominantly colonized by *Lactobacillus* that maintains a protective microenvironment. Microbial disruption and subsequent flora abnormality promote carcinogenesis via chronic inflammation, immune dysfunction, and metabolic dysregulation (Javadi et al., 2024).

In endometrial cancer, distinct endometrial microbial profiles have been documented, and endometrial dysbiosis is tightly associated with carcinogenesis through persistent inflammation and dysregulated immune responses (Gong et al., 2025). A systematic review by Aquino et al. confirmed that microbial dysbiosis facilitates endometrial tumorigenesis primarily via sustained local inflammation (Aquino et al., 2024). Overall, microbial dysbiosis represents a convergent pathogenic mechanism across female reproductive tract malignancies, providing promising targets for microbiota-based prevention and treatment strategies (Chalif et al., 2024).

This review is organized along a logical translational chain: we begin by summarizing the epidemiological evidence linking vaginal dysbiosis to HPV and cervical lesions (Section 1), then discuss the proposed immune, inflammatory and metabolic mechanisms that may explain these associations (Section 2). Next, we examine microbial metabolites as potential mediators and biomarkers (Section 3), followed by a critical evaluation of current diagnostic technologies and microbiome-modulating interventions (Sections 4 and 5). Finally, we highlight ongoing controversies, technical challenges, and priorities for future research (Section 6). Within each section, we distinguish findings from meta-analyses or well-controlled longitudinal cohorts from those derived from smaller, exploratory or cross-sectional studies.

## 2 Basic research on the vaginal microbiome in cervical cancer patients

### 2.1 Composition of the vaginal microbiota and its relationship with cervical cancer

The vaginal microbiota is a critical factor in maintaining female reproductive health, and its dysbiosis is closely associated with cervical carcinogenesis. Under normal physiological conditions, the vaginal microbiota is dominated by *Lactobacillus* species (e.g., *L. crispatus, L. iners*), which produce lactic acid to maintain an acidic environment (pH < 4.5) that inhibits pathogen colonization (Audirac-Chalifour et al., 2016). However, the vaginal microbiota in cervical cancer patients exhibits marked alterations: a cross-sectional study involving 90 women demonstrated that, relative to healthy controls, patients exhibited a significant decrease in *Lactobacillus* abundance (*P* < 0.05) alongside significantly elevated abundances of pathogenic bacteria such as *Prevotella* spp., *Sneathia* spp., and *Pseudomonas* spp. (Xie et al., 2020). Further research indicates that HPV-positive women harbor a significantly more diverse vaginal microbiota than HPV-negative women (*P* < 0.05), with microbial diversity progressively increasing with the severity of cervical lesions—from normal tissue through low-grade squamous intraepithelial lesion (LSIL) and high-grade squamous intraepithelial lesion (HSIL) to invasive cervical cancer (Mitra et al., 2016; Brusselaers et al., 2019). For instance, in a small study of 59 Chinese women, the microbial α-diversity (Chao1 index) in the cervical cancer group was significantly elevated compared to the normal group (*P* = 0.046), with a marked enrichment of the Actinobacteria phylum in diseased groups (Wei et al., 2022). Specific bacterial genera have been implicated: *Fusobacterium* spp. predominate in cervical cancer patients, with their abundance positively correlated to the mRNA levels of immunosuppressive factors like IL-4 and TGF-β1 (Audirac-Chalifour et al., 2016); *Sneathia* spp. are enriched in HSIL and cancer patients and show significant associations with high-risk HPV types such as HPV16 and HPV58 (Łaniewski et al., 2018; Zhang et al., 2018). These findings suggest that compositional shifts in the vaginal microbiota may be associated with persistent HPV infection and cervical lesion progression by modulating the local immune microenvironment.

The roles of distinct *Lactobacillus* species in cervical carcinogenesis appear to diverge. *L. crispatus* is considered the most protective species, with decreased abundance significantly correlated with increased risks of HPV infection and cervical lesions (OR = 0.49, 95% CI: 0.31–0.79) (Wang et al., 2019). In contrast, while *L. iners* is a common vaginal commensal, its abundance shows no significant protective association and may, under certain conditions, even may contribute to disease progression (Brusselaers et al., 2019; Wei et al., 2022). A study on Korean women, for example, found a positive correlation between *L. iners* abundance and cervical cancer severity, hinting at its potential utility as a biomarker for disease progression (Wei et al., 2022). The synergistic interplay between the vaginal microbiota and HPV infection has also garnered attention: among female sex workers in Kenya, bacterial vaginosis (BV) was significantly associated with HPV58 infection (aOR = 2.3, 95% CI: 1.0–5.2), while Candida infection was significantly associated with HPV16 (aOR = 1.9, 95% CI: 1.1–3.3) (Menon et al., 2016). These observations provide crucial context for understanding the microbiota's role in cervical cancer, though the precise underlying mechanisms require further elucidation.

### 2.2 Dynamic changes of the vaginal microbiota in cervical cancer progression

The dynamic nature of the vaginal microbiota is intimately tied to cervical cancer progression, with diversity and composition undergoing substantial shifts corresponding to lesion grade. A longitudinal pilot study revealed that from normal cervix through cervical intraepithelial neoplasia (CIN) to cervical cancer, α-diversity progressively increased, while β-diversity exhibited the greatest within-group variance in the cancer cohort (*P* < 0.0006) (Audirac-Chalifour et al., 2016). In a study of 49 cervical cancer patients, pre-treatment microbiota was predominantly characterized by Prevotella, exhibiting high diversity with only 20% of samples being *Lactobacillus*-dominant; post-treatment, patients with persistent HPV infection displayed significantly higher microbial diversity than those who cleared the virus, and Prevotella abundance was significantly associated with cancer recurrence (*P* < 0.05) (Tsementzi et al., 2025). Furthermore, shifts in the microbiota correlate with HPV infection status: patients with persistent HPV infection demonstrated a significantly higher rate of community state type conversion (e.g., from *Lactobacillus*-dominant to non-*Lactobacillus*-dominant) compared to HPV clearers (34% vs. 19%, *P* = 0.036) (Berggrund et al., 2020).

The dynamics of specific microbial species in relation to disease progression are under intensive investigation. For instance, Streptococcus vaginalis, detected in 27% of Korean women studied, induces oxidative stress through hydrogen peroxide production, activating the endoplasmic reticulum unfolded protein response (UPR) and thereby affecting proliferation, migration, and apoptosis of cervical cancer cells (Montecillo et al., 2026). Another study found that *Lactobacillus iners* can induce metabolic reprogramming via L-lactate production, conferring chemoradiation resistance on cervical cancer cells (Colbert et al., 2023). Additionally, the gut microbiota may influence cervical cancer progression through its metabolites: a study involving 578 specimens indicated that gut microbial taxa like *Allisonella* and *Lachnospiraceae* were significantly associated with HPV types, with superior predictive accuracy for CIN grading (85.52%) compared to the vaginal microbiota alone (83.33%) (Meng et al., 2026). These results collectively imply that the dynamic evolution of both vaginal and gut microbial communities may not merely be a consequence of, but also a contributing factor to, lesion progression, offering novel targets for early intervention in cervical cancer.

### 2.3 Pathological mechanisms of the vaginal microbiome in cervical cancer

The vaginal microbiome engages in cervical carcinogenesis through multiple mechanisms, primarily encompassing immunomodulation, inflammatory responses, metabolic reprogramming, and DNA damage. Firstly, microbial dysbiosis can induce localized immunosuppression: a *Fusobacterium* spp.-dominated microbiota significantly upregulates the expression of immunosuppressive factors such as IL-4 and TGF-β1, thereby dampening host anti-tumor immune responses (Audirac-Chalifour et al., 2016). Secondly, pathogenic bacteria such as *Gardnerella vaginalis* and *Atopobium vaginae* can produce metabolites like lipopolysaccharide (LPS) and short-chain fatty acids (SCFAs) that activate signaling pathways such as NF-κB and PI3K/Akt, fostering inflammation and cellular proliferation (Huang et al., 2024; Sun et al., 2025). For example, one study revealed that HPV infection can downregulate the SLC25A26 gene, leading to defective mitochondrial SAM transport, subsequently triggering mtDNA hypermethylation and metabolic reprogramming to favor tumor cell survival (Menga et al., 2017).

Microbial metabolites have been proposed to play a crucial role in the pathological mechanisms of cervical cancer. D-lactate produced by *Lactobacillus* species has been reported to promote wound healing via M2 macrophage polarization and block HPV infection (Peng et al., 2025); conversely, butyrate generated by *Fusobacterium* spp. can promote HPV E6/E7 oncogene expression by inhibiting histone deacetylases (HDACs) (Huang et al., 2024). Moreover, the microbiota can contribute to cervical cancer through oxidative stress modulation: hydrogen peroxide secreted by Streptococcus vaginalis induces endoplasmic reticulum stress in cervical cancer cells, activating the UPR pathway and leading to apoptosis (Montecillo et al., 2026); meanwhile, erucic acid produced by *Lactobacillus crispatus* activates the PPAR-δ signaling pathway, promoting fatty acid oxidation and reactive oxygen species (ROS) generation to induce ferroptosis in cervical cancer cells (Zhen et al., 2025). The elucidation of these mechanisms provides a theoretical basis for targeted therapy in cervical cancer, though further *in vivo* validation remains essential.

## 3 Association of vaginal microbial metabolites with cervical cancer

In this section, we focus primarily on metabolites that are produced by or derived from the vaginal microbiota. For completeness, we also briefly mention some host- or drug-related metabolic pathways (e.g., miR-22, cisplatin) that are not microbiome-derived but have been studied in the context of cervical cancer metabolism; these are included only for context and are not the main focus of this review.

### 3.1 Mechanisms of action of metabolites in cervical cancer

Vaginal microbial metabolites influence cervical cancer development and progression through diverse pathways, notably including metabolic reprogramming of energy pathways, signaling pathway regulation, and modulation of the immune microenvironment. Firstly, metabolites may contribute to tumor cell proliferation by modulating glycolysis: the HPV E6 protein can downregulate miR-34a, thus lifting inhibition of lactate dehydrogenase A (LDHA) and enhancing the Warburg effect with concomitant increased lactate production (*P* < 0.05) (Zhang et al., 2016). Secondly, metabolites can influence tumor progression via lipid metabolism regulation: miR-22 directly targets ATP citrate lyase (ACLY) to inhibit *de novo* lipid synthesis, thereby suppressing proliferation and invasion of cervical cancer cells (*P* < 0.05) (Xin et al., 2016). Metabolites also participate in cervical carcinogenesis through amino acid metabolism: tryptophan catabolites like kynurenine can activate the AhR signaling pathway, inhibiting T-cell function and facilitating immune escape (Ilhan et al., 2019).

Interactions between microbial metabolites and host cells are also integral to cervical cancer pathogenesis. As discussed in Section 2(3), specific metabolites—such as D-lactate and butyrate—have been shown to influence cervical cancer cell behavior through receptor-mediated and epigenetic mechanisms. Additionally, metabolites participate in cervical cancer progression by affecting oxidative stress: vitamin K3 can induce ROS production in cervical cancer cells, resulting in decreased mitochondrial membrane potential and apoptosis (*P* < 0.05) (de Carvalho Scharf Santana et al., 2016); niclosamide can enhance the sensitivity of cervical cancer cells to paclitaxel by inhibiting the mTOR signaling pathway (*P* < 0.05) (Chen et al., 2017). Research into these mechanisms provides fresh perspectives for metabolic interventions in cervical cancer.

### 3.2 Metabolite detection techniques and cervical cancer diagnosis

Metabolite detection technologies hold significant promise for cervical cancer diagnosis, principally including mass spectrometry (MS), nuclear magnetic resonance (NMR) spectroscopy, and imaging modalities. Firstly, liquid chromatography-mass spectrometry (LC-MS) can be employed to detect metabolites in cervicovaginal secretions: a study involving 78 patients identified 3-hydroxybutyrate, eicosenoic acid, and oleic/vaccenic acid as preliminary potential diagnostic biomarkers for cervical cancer, yielding area under the curve (AUC) values of 0.92, 0.89, and 0.87, respectively (Ilhan et al., 2019). Secondly, direct injection electrospray ionization mass spectrometry (DI-ESI-MS) enables rapid profiling of cervical cell metabolites: a double-blind study demonstrated that this technique could distinguish between individuals with persistent HPV infection and those who cleared the virus based on metabolite profiles, achieving 85% accuracy (Walker et al., 2017). Furthermore, integrated metabolomic and transcriptomic analysis can improve diagnostic accuracy: a study including 285 patients found that a combined bilirubin, acyl-carnitine, and lipid metabolite panel achieved high diagnostic accuracy for distinguishing cervical cancer from normal controls, with AUC values reaching 0.98 and 0.99 (Yang et al., 2017).

Imaging technologies also offer potential in metabolite detection. Positron emission tomography (PET) can aid cervical cancer diagnosis by assessing tumor glucose metabolism levels: a study of 32 patients revealed that the standardized uptake value (SUV) of tumor tissue correlated with pathological grade, albeit without statistical significance (*P* > 0.05) (Mocciaro et al., 2016). Magnetic resonance spectroscopy (MRS) can detect metabolites within cervical tissue: a study of 87 patients showed that a scoring system combining diffusion-weighted imaging (DWI) and MRS achieved 93% accuracy in diagnosing cervical adenocarcinoma (Lin et al., 2018). Additionally, volatile organic compound (VOC) detection can be applied to cervical cancer diagnosis: one study found that cervical cancer cells produce specific VOCs detectable via gas chromatography-mass spectrometry (GC-MS) with 89% accuracy, 93% sensitivity, and 93% specificity (Rodríguez-Esquivel et al., 2018). While these technological advances provide novel means for early cervical cancer detection, they require further standardization.

### 3.3 Potential of metabolites as therapeutic targets in cervical cancer

While most of the following examples involve host- or drug-related metabolic pathways, they illustrate the broader concept of metabolic targeting in cervical cancer and are included here to contextualize the potential relevance of microbiome-derived metabolites, which remain less extensively studied. Metabolites hold considerable promise as therapeutic targets in cervical cancer, including inhibitors of energy metabolism, modulators of lipid metabolism, and amino acid metabolism intervention agents. Firstly, energy metabolism inhibitors such as cisplatin can reduce glucose uptake and lactate production in cervical cancer cells by suppressing integrin β5-mediated glycolysis (*P* < 0.05) (Wang et al., 2016); doxycycline can induce apoptosis in cervical cancer cells by inhibiting mitochondrial respiration and ATP generation (*P* < 0.05) (Zhao et al., 2016). Secondly, lipid metabolism modulators like miR-22 can inhibit lipid synthesis and proliferation of cervical cancer cells by targeting ACLY (*P* < 0.05) (Xin et al., 2016); tetrahydrocurcumin can suppress cervical cancer cell proliferation by inhibiting COX-2, EGFR, and other signaling pathways (*P* < 0.05) (Yoysungnoen et al., 2016). Thirdly, amino acid metabolism intervention agents like vitamin K3 can inhibit cervical cancer cell proliferation by inducing ROS generation (*P* < 0.05) (de Carvalho Scharf Santana et al., 2016); niclosamide can enhance paclitaxel sensitivity in cervical cancer cells by increasing oxidative stress (*P* < 0.05) (Chen et al., 2017).

Targeting microbial metabolite pathways represents a promising therapeutic strategy. For instance, the D-lactate/GPR81 axis has been proposed to suppress tumor cell proliferation, while butyrate-mediated HDAC inhibition has been linked to enhanced HPV oncogene expression, making butyrate production inhibition a potential therapeutic strategy (for detailed mechanistic discussion, see Section 2(3). Moreover, integrated metabolomic and microbiome analyses can reveal novel therapeutic targets: one study showed that glycerophospholipid metabolites, such as PE(18:1/0:0), were significantly correlated with Prevotella bivia abundance, suggesting their potential as intervention targets (Pu et al., 2025). These findings chart new directions for metabolic therapy in cervical cancer, though further clinical validation remains necessary.

## 4 Epidemiological studies on the vaginal microbiome in cervical cancer patients

### 4.1 Epidemiological characteristics of the vaginal microbiota and cervical cancer

Dysbiosis of the vaginal microbiota is intricately linked to the epidemiological features of cervical cancer, primarily manifesting as heightened microbial diversity, decreased *Lactobacillus* abundance, and enrichment of pathogenic bacteria. A meta-analysis encompassing 101,049 women provides the strongest current evidence, revealed that vaginal dysbiosis was significantly associated with an increased risk of HPV infection (RR = 1.33, 95% CI: 1.18–1.50), persistent HPV infection (RR = 1.14, 95% CI: 1.01–1.28), and high-grade lesions and cervical cancer (RR = 2.01, 95% CI: 1.40–3.01) (Brusselaers et al., 2019). Furthermore, bacterial vaginosis (BV) is significantly linked to persistent HPV infection (OR = 2.15, 95% CI: 1.13–4.08) (Kero et al., 2017), and Trichomonas vaginalis infection synergizes with HPV16 infection, increasing the risk of CIN 2–3 (OR = 1.71, 95% CI: 1.16–2.53) (Yang et al., 2020).

The characteristics of the vaginal microbiota vary across geographic regions. For example, a non-*Lactobacillus*-dominant microbiota is substantially more prevalent among African women compared to their European counterparts (56.5% vs. 20%) (Onywera et al., 2019); Latina women exhibit significantly higher *Lactobacillus iners* abundance than non-Latina women, which correlates with increased HPV infection risk (Łaniewski et al., 2018). Moreover, specific populations, such as female sex workers, display more pronounced dysbiosis: among Kenyan female sex workers, BV prevalence reached 48.3%, *T. vaginalis* infection rate was 31.4%, both of which were significantly associated with high-risk HPV types like HPV58 and HPV16 (Menon et al., 2016). These epidemiological characteristics inform precision prevention strategies, yet further cross-regional studies are needed for validation.

### 4.2 Differences in vaginal microbiota and cervical cancer across populations

Significant inter-population differences exist in the association between the vaginal microbiota and cervical cancer, influenced primarily by race, age, and lifestyle factors. First, regarding race: African American women tend to have higher vaginal microbial diversity compared to white women, along with lower *Lactobacillus crispatus* abundance, which correlates with an increased risk of cervical cancer (OR = 7.8, 95% CI: 1.7–74.5) (Tossas et al., 2023); in contrast, Latina women have higher *Lactobacillus* iners abundance than non-Latina women, associated with higher HPV infection risk (Łaniewski et al., 2018). Second, regarding age: younger women (18–25 years) exhibit higher vaginal microbial diversity, correlating with increased HPV infection risk (RR = 1.43, 95% CI: 1.10–1.85) (Brusselaers et al., 2019); postmenopausal women often harbor a non-*Lactobacillus*-dominant microbiota, linked to an elevated risk of cervical cancer (Cocomazzi et al., 2024). Third, regarding lifestyle: vaginal douching can significantly reduce *Lactobacillus* abundance and increase HPV infection risk (OR = 2.75, 95% CI: 1.77–4.27) (Aslan and Bechelaghem, 2018); conversely, hormonal contraceptive use may increase *Lactobacillus iners* abundance and reduce HPV infection risk (OR = 0.125, 95% CI: 0.02–0.65) (van de Wijgert et al., 2020).

Differences also emerge in the associations between specific HPV types and the vaginal microbiota. For instance, HPV16 infection is significantly associated with Candida infection (aOR = 1.9, 95% CI: 1.1–3.3) (Menon et al., 2016); HPV58 infection is significantly associated with BV (aOR = 2.3, 95% CI: 1.0–5.2) (Menon et al., 2016). Persistent HPV infection is related to the dynamics of the microbiota: individuals with persistent HPV show a significantly higher conversion rate of their microbiota community state type than those who clear the infection (34% vs. 19%, *P* = 0.036) (Berggrund et al., 2020). These variations provide a foundation for individualized prevention and treatment of cervical cancer, though larger-scale studies are needed for confirmation.

### 4.3 Association between the vaginal microbiome and cervical cancer risk factors

The vaginal microbiome is closely associated with established cervical cancer risk factors, including HPV infection, sexually transmitted infections (STIs), immune status, and lifestyle habits. First, HPV infection: vaginal dysbiosis is significantly linked to an increased risk of HPV infection (RR = 1.33, 95% CI: 1.18–1.50) (Brusselaers et al., 2019), with specific genera such as *Gardnerella vaginalis* notably associated with persistent HPV infection (OR = 2.15, 95% CI: 1.13–4.08) (Kero et al., 2017). Second, STIs: *T. vaginalis* infection synergistically increases CIN 2–3 risk among HPV16-positive individuals (OR = 1.71, 95% CI: 1.16–2.53) (Yang et al., 2020); Chlamydia trachomatis infection is significantly correlated with increased vaginal microbial diversity (*P* < 0.05) (Di Pietro et al., 2018). Third, immune status: HIV-positive women exhibit significantly higher vaginal microbial diversity than HIV-negative women, correlating with an increased risk of persistent HPV infection (OR = 3.28, 95% CI: 1.31–8.74) (Adebamowo et al., 2017); IBD patients undergoing immunosuppressive therapy show a significantly elevated prevalence of BV (*P* < 0.05) (Ricci et al., 2020).

Lifestyle habits also modulate the relationship between the vaginal microbiome and cervical cancer risk. For example, vaginal douching significantly reduces *Lactobacillus* abundance and raises HPV infection risk (OR = 2.75, 95% CI: 1.77–4.27) (Aslan and Bechelaghem, 2018); hormonal contraceptive use may elevate *Lactobacillus iners* abundance and lower HPV infection risk (OR = 0.125, 95% CI: 0.02–0.65) (van de Wijgert et al., 2020). In addition, smoking is significantly correlated with *T. vaginalis* infection (OR = 3.18, 95% CI: 1.23–8.24) (da Silva Pinto et al., 2023), consequently potentially increasing cervical cancer risk. These associations provide a basis for risk assessment, but longitudinal studies are still required to verify causal relationships.

**Figure 1:**
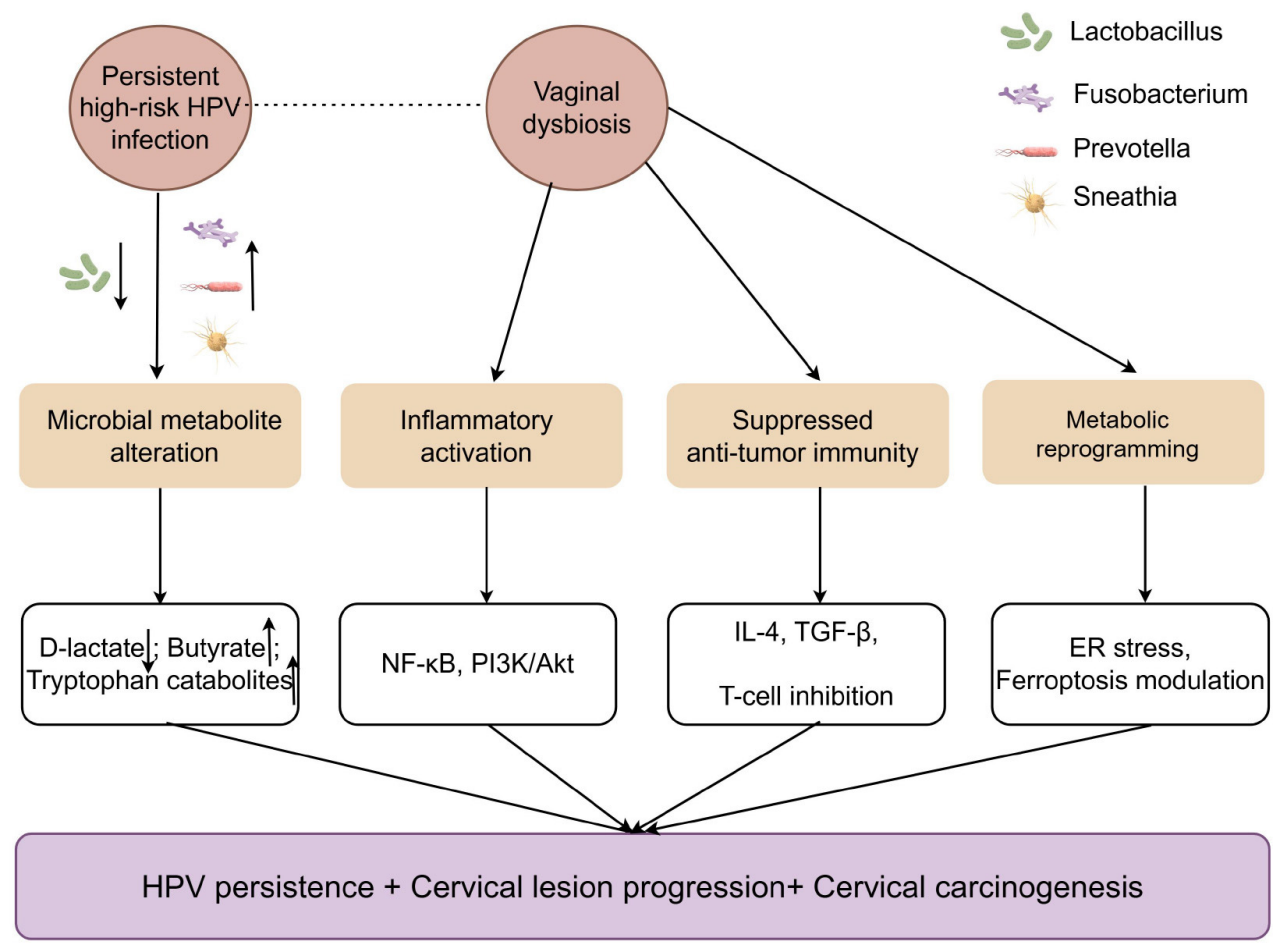
Schematic diagram of proposed interactions among HPV, vaginal dysbiosis, and cervical carcinogenesis. Persistent high-risk HPV infection is associated with a shift from Lactobacillus-dominant to a more diverse, anaerobe-enriched microbiota (e.g., *Fusobacterium, Prevotella, Sneathia*). These compositional changes may lead to altered production of microbial metabolites (decreased D-lactate, increased butyrate and tryptophan catabolites), which in turn may activate inflammatory pathways (NF-κB, PI3K/Akt), suppress local anti-tumor immunity (IL-4, TGF-β, *T*-cell inhibition), and induce metabolic reprogramming (ER stress, ferroptosis modulation)—collectively contributing to HPV persistence and lesion progression. Dashed arrows indicate proposed relationships that require further mechanistic validation.

To facilitate the reader‘s overview of the field, Table 1 summarizes selected key studies discussed in this review, highlighting study design, sample size, main findings, and the strength of evidence. Figure 1 presents a conceptual model integrating the main pathways through which vaginal dysbiosis and microbial metabolites may influence HPV persistence and cervical lesion progression.

**Table 1:** Key studies on vaginal microbiome and metabolites in cervical cancer.

References	Study design	Sample size	Key finding	Evidence level
Brusselaers et al. (2019)	Meta-analysis	101,049	Dysbiosis associated with HPV (RR 1.33), persistence (RR 1.14), cancer (RR 2.01)	Strong
Wang et al. (2019)	Meta-analysis	>10,000	*L. crispatus* protective (OR 0.49); *L. iners* not protective	Strong
Xie et al. (2020)	Cross-sectional	59	↑α-diversity in cancer; enrichment of Actinobacteria	Preliminary
Ilhan et al. (2019)	Cross-sectional	78	3-hydroxybutyrate AUC 0.92 as biomarker	Preliminary
Tsementzi et al. (2025)	Longitudinal	49	*Prevotella* linked to recurrence	Moderate
Dellino et al. (2022)	RCT (open-label)	Not specified	*L. crispatus* M247 improved HPV clearance	Moderate

## 5 Application of vaginal microbiome in cervical cancer diagnostic techniques

In this section, we discuss both clinically established and emerging diagnostic approaches. We distinguish between: (i) clinically validated methods (HPV DNA testing and cytology, currently guideline-recommended); (ii) promising but investigational technologies (16S sequencing-based and metabolomic biomarkers, requiring further validation); and (iii) proof-of-concept or experimental platforms (nanopore sequencing, CRISPR assays, AI-assisted models, microfluidics and wearables).

### 5.1 Application of vaginal microbial detection in early cervical cancer screening

Vaginal microbial detection holds substantial value in early cervical cancer screening, with primary techniques including 16S rRNA sequencing, multiplex PCR, and metabolomic analysis. First, 16S rRNA sequencing can profile the vaginal microbial composition: a study of 272 women identified increased abundance of genera such as Stenotrophomonas and Streptococcus as significantly associated with HSIL, where the predictive model achieved an AUC of 0.952 (Chao et al., 2021). Second, multiplex PCR enables concurrent detection of HPV and microbial biomarkers: one assay developed could simultaneously detect 14 high-risk HPV types, 5 low-risk HPV types, and 31 bacterial taxa, achieving 94.5% sensitivity and 96.6% specificity for high-risk HPV detection (Bik et al., 2019). Third, metabolomic profiling can be utilized for early screening: a study of 78 patients identified metabolites like 3-hydroxybutyrate and eicosenoic acid as potential early diagnostic biomarkers for cervical cancer, with an AUC reaching 0.92 (Ilhan et al., 2019).

The adoption of self-sampling techniques has improved screening accessibility. A study indicated that the accuracy of HPV testing on self-collected samples is comparable to clinician-collected samples, significantly increasing participation rates among previously unscreened women (*P* < 0.05) (Gupta et al., 2018). Furthermore, self-collected samples can be simultaneously processed for microbiota analysis: a newly developed assay can concurrently detect HPV, STIs, and the microbiota from a single self-sample, with 95.3% agreement for high-risk HPV detection (Bik et al., 2019). These technological applications provide new tools for early cervical cancer screening, yet broader implementation is needed.

### 5.2 Value of vaginal microbial biomarkers in cervical cancer diagnosis

Vaginal microbial biomarkers are valuable in cervical cancer diagnosis, encompassing specific bacterial genera, metabolites, and community diversity indices. First, specific bacterial genera: *Fusobacterium* spp. abundance is significantly elevated in cervical cancer patients (*P* < 0.05) and positively correlated with the expression of immunosuppressive factors like IL-4 and TGF-β1 (Audirac-Chalifour et al., 2016); *Sneathia* spp. is enriched in patients with HSIL and cervical cancer, with its abundance significantly associated with HPV16 infection (*P* < 0.05) (Łaniewski et al., 2018). Second, metabolites: 3-hydroxybutyrate and sphingolipids can serve as diagnostic biomarkers for cervical cancer, with AUC values of 0.92 and 0.89, respectively (Ilhan et al., 2019); VOCs such as alkanes, detectable via GC-MS, yield a diagnostic accuracy of 89% (Rodríguez-Esquivel et al., 2018). Third, microbial diversity: the vaginal microbiota of cervical cancer patients displays significantly increased α-diversity compared to healthy controls (*P* < 0.05), alongside marked β-diversity differences (Audirac-Chalifour et al., 2016).

Combining multiple microbial biomarkers can improve diagnostic accuracy. For example, a diagnostic model incorporating *Lactobacillus crispatus* abundance, *Gardnerella vaginalis* abundance, and microbial diversity achieved an AUC of 0.982 (Kang et al., 2021); another model integrating metabolites and microbiome data reached an AUC of 0.99 (Lin et al., 2018). Furthermore, microbial biomarkers can predict lesion progression: a study reported that *Gardnerella vaginalis* abundance was significantly associated with progression from high-risk HPV infection to CIN2+ (OR = 1.19, 95% CI: 1.03–1.38) (Usyk et al., 2020). The application of these biomarkers underpins precision diagnostics for cervical cancer, though additional clinical validation is required.

### 5.3 Future prospects of novel diagnostic technologies for vaginal microbiome application

Novel diagnostic technologies, including nanopore sequencing, CRISPR-based diagnostics, and artificial intelligence (AI)-assisted (research use only, not clinically validated) analysis, offer new frontiers for applying the vaginal microbiome in cervical cancer diagnosis. First, nanopore sequencing enables real-time detection: a study demonstrated that microbiome analysis results obtained via nanopore sequencing showed 92% concordance with Illumina sequencing, permitting rapid detection of HPV and microbial biomarkers (Graeber et al., 2025). Second, CRISPR-based diagnostics (proof-of-concept stage) can enhance detection sensitivity: CRISPR-based diagnostics have been proposed for point-of-care applications; as proof of concept, a CRISPR-Cas12a assay has demonstrated sensitivity of 100 copies/mL for HPV16/18 in laboratory settings (Ghouneimy et al., 2023). Third, AI-assisted analysis can improve diagnostic accuracy: a diagnostic model built using the random forest algorithm predicted cervical cancer with an AUC of 0.913 (Kang et al., 2020); another AI-integrated metabolomic model reached an AUC of 0.99 (Yang et al., 2017).

Microfluidic technology also offers new avenues for vaginal microbial testing. A developed microfluidic chip designed for tumor-endothelial interaction studies demonstrates the feasibility of rapid multi-parameter detection in research settings, and similar principles could potentially be adapted for HPV and microbiota detection (Lin et al., 2017). Additionally, wearable devices (speculative, preclinical concept) enable real-time monitoring: a vaginal sensor developed for continuous monitoring of pH, microbiota, and metabolites has been proposed for gynecological applications, though its use specifically for cervical cancer remains speculative (Liu et al., 2019). The advancement of these novel technologies presents broad prospects for the diagnostic application of the vaginal microbiome in cervical cancer, though further optimization is still required.

## 6 Vaginal microbiome modulation in cervical cancer therapeutic strategies

Therapeutic microbiome modulation ranges from relatively established interventions (antibiotics for BV, which are guideline-supported for BV itself, though not specifically for cervical cancer) through promising but still investigational approaches (probiotics, with mixed trial results), to highly experimental strategies (VMT, metabolite-based adjuvants). The following discussion reflects this gradient of clinical readiness.

### 6.1 Modulation of the vaginal microbiota and cervical cancer treatment

Modulating the vaginal microbiota plays a significant role in cervical cancer treatment, primarily through antibiotic therapy, probiotic intervention (investigational, with mixed trial results), and microbiota transplantation. First, antibiotic therapy can eradicate pathogenic bacteria: one study showed that treating BV with metronidazole significantly reduced the risk of persistent HPV infection (OR = 0.5, 95% CI: 0.3–0.8) [34]; another demonstrated that treating aerobic vaginitis with rifaximin significantly improved vaginal microbial composition (*P* < 0.0001) (Donders et al., 2017). Second, probiotic intervention can restore *Lactobacillus* abundance: the use of *Lactobacillus crispatus* probiotics was shown to significantly enhance HPV clearance rates (OR = 0.41, 95% CI: 0.22–0.79) (Wang et al., 2019); *Lactobacillus rhamnosus* probiotics substantially reduced the incidence of radiotherapy-induced diarrhea (53.8% vs. 82.1%, *P* < 0.05) (Linn et al., 2019). Third, vaginal microbiota transplantation (VMT) (experimental, only pilot data available) can rehabilitate the vaginal ecosystem: one study found that VMT significantly improved the BV cure rate (80% vs. 20%, *P* < 0.05) (Yockey et al., 2022).

Microbiota modulation can also potentiate the efficacy of chemoradiotherapy. For example, probiotic use significantly increased the sensitivity of cervical cancer cells to cisplatin (*P* < 0.05) (Negi et al., 2020); antibiotic-mediated clearance of pathogenic bacteria markedly enhanced radiotherapy effectiveness (*P* < 0.05) (Karpinets et al., 2020). Additionally, microbial metabolites can act as therapeutic adjuvants. Moreover, the potential involvement of microbial metabolites such as butyrate in modulating HPV oncogene expression—as detailed in Section 2(3)—suggests that targeting metabolite production may influence recurrence risk. These strategies present new avenues for combinatorial cervical cancer therapy, though further clinical research is warranted.

### 6.2 Research on probiotic applications in cervical cancer treatment

Research on probiotics in cervical cancer treatment primarily focuses on HPV clearance, alleviation of chemoradiation side effects, and immunomodulation. First, regarding HPV clearance: oral administration of *Lactobacillus crispatus* M247 significantly improved HPV clearance rates (15.3% vs. 9.3%, *P* < 0.05) (Dellino et al., 2022); *Lactobacillus rhamnosus* probiotics significantly decreased the risk of persistent HPV infection (OR = 0.5, 95% CI: 0.3–0.8) (Pourmollaei et al., 2020). Second, concerning side effect mitigation: a meta-analysis demonstrated that probiotics significantly reduced the incidence of radiotherapy-induced diarrhea (RR = 0.61, 95% CI: 0.46–0.81) (Qiu et al., 2019); probiotic supplementation considerably lowered the incidence of chemotherapy-induced nausea and vomiting (*P* < 0.05) (Negi et al., 2020). Third, in terms of immunomodulation: Bifidobacterium bifidum probiotics significantly boosted tumor-specific IFN-γ and CD8+ T-cell responses (*P* < 0.05) (Abdolalipour et al., 2020); *Lactobacillus gasseri* probiotics significantly upregulated IL-10 expression, dampening inflammatory reactions (*P* < 0.05) (Nicolò et al., 2021).

The mechanisms of probiotic action mainly involve competitive inhibition, immunomodulation, and the effects of their metabolites. For instance, *Lactobacillus crispatus* produces lactic acid to lower vaginal pH, thereby inhibiting pathogen growth (Peng et al., 2025); Bifidobacterium bifidum can activate the TLR4 signaling pathway to enhance immune responses (Abdolalipour et al., 2020). These mechanistic insights provide a theoretical foundation for probiotic application in cervical cancer treatment, but more clinical studies are required for verification.

### 6.3 Impact of vaginal microbiome interventions on cervical cancer prognosis

Vaginal microbiome interventions have a notable impact on cervical cancer prognosis, chiefly in terms of HPV clearance, reduced recurrence risk, and improved quality of life. First, HPV clearance: a study found that probiotics significantly increased HPV clearance rates (15.3% vs. 9.3%, *P* < 0.05) [60]; antibiotic treatment for BV significantly lowered the risk of persistent HPV infection (OR = 0.5, 95% CI: 0.3–0.8) (Kero et al., 2017). Second, reduced recurrence risk: imbalance in the vaginal microbiome was significantly associated with cervical cancer recurrence (OR = 3.789, 95% CI: 1.091–13.154) (Ma et al., 2024); administration of probiotics significantly decreased recurrence risk (OR = 0.41, 95% CI: 0.22–0.79) (Wang et al., 2019). Third, improved quality of life: probiotics significantly reduced the incidence of radiotherapy-induced diarrhea (53.8% vs. 82.1%, *P* < 0.05) (Linn et al., 2019); VMT significantly alleviated vaginal discomfort symptoms (*P* < 0.05) (Yockey et al., 2022).

The mechanisms underlying the prognostic impact of microbial interventions mainly involve immunomodulation, metabolic reprogramming, and suppression of inflammation. For example, *Lactobacillus crispatus* has been reported to exert antiproliferative effects via D-lactate-mediated pathways (see Section 2(3) for mechanistic details); Bifidobacterium bifidum can suppress tumor recurrence by boosting CD8+ T-cell responses (*P* < 0.05) (Abdolalipour et al., 2020). Moreover, microbial metabolites like butyrate may contribute to HPV E6/E7 expression through HDAC inhibition, suggesting that reducing butyrate production may lower recurrence risk (*P* < 0.05) (Huang et al., 2024). These mechanistic studies provide a theoretical groundwork for the prognostic utility of vaginal microbiome interventions in cervical cancer, though more clinical validation is imperative.

## 7 Controversies and challenges in research on the vaginal microbiome and cervical cancer

### 7.1 Controversial points in vaginal microbiome research in cervical cancer

Controversies surrounding the role of the vaginal microbiome in cervical cancer research primarily involve causality, the definition of a ‘healthy' microbiota, and treatment efficacy. First, regarding causality: although numerous studies indicate an association between vaginal dysbiosis and cervical cancer, direct causal evidence remains sparse. For instance, one study indicated that HPV infection could lead to an imbalance in the vaginal microbiota (OR = 3.246, 95% CI: 1.07–9.82) (Zhang et al., 2024), whereas another suggested that dysbiosis promotes persistent HPV infection (OR = 2.15, 95% CI: 1.13–4.08) (Kero et al., 2017), hinting at a potential bidirectional relationship. Second, concerning the definition of ‘healthy' microbiota: definitions vary across studies. While some consider an *L. iners*-dominant microbiota as healthy, others link it to lesion progression (Brusselaers et al., 2019; Mocciaro et al., 2016). Third, treatment efficacy: results from probiotic intervention trials are inconsistent, with some demonstrating a significant improvement in HPV clearance (*P* < 0.05) (Dellino et al., 2022) while others report no significant effect (*P* > 0.05) (Bik et al., 2019; Sasidharan et al., 2019).

These controversies stem largely from heterogeneous study designs, insufficient sample sizes, and differing detection technologies. Cross-sectional studies, for example, cannot determine causality, while longitudinal studies often suffer from small sample sizes; different analytical technologies (e.g., 16S rRNA sequencing vs. culture-based methods) introduce variances in microbiome analysis results (Ho et al., 2021). Additionally, population heterogeneity contributes to discrepant findings, as vaginal microbiome characteristics vary by race, age, and lifestyle (Onywera et al., 2019; Tossas et al., 2023). These unresolved issues chart the course for future research, underscoring the need for large-scale longitudinal studies and standardized detection techniques.

### 7.2 Technical challenges and solutions in vaginal microbiome research

Technical challenges in vaginal microbiome research include specimen contamination, lack of standardization in detection, and complex data analysis. First, regarding contamination: vaginal specimens are susceptible to contamination from skin, urethral, or gut microbiota, leading to biased results. Solutions include stringent specimen collection protocols (e.g., using sterile swabs, minimizing contact) and bioinformatic decontamination methods (e.g., removing low-abundance OTUs, employing negative controls) (Ho et al., 2021). Second, concerning standardization: the choice of hypervariable region for 16S rRNA gene sequencing (e.g., V3–V4 vs. V4–V5) can influence taxonomic profiling outcomes (Ho et al., 2021). Solutions involve adopting a consistent sequencing region, standardized library preparation protocols, and a unified reference database (Vieira-Baptista et al., 2022). Third, regarding data analysis: microbiome data is high-dimensional and inherently noisy, posing challenges for conventional statistical approaches. Solutions include leveraging machine learning algorithms (e.g., random forest, support vector machines) and multi-omics integration strategies (Ogundemuren et al., 2026).

Emerging technologies offer potential solutions to these challenges. For instance, nanopore sequencing facilitates real-time detection while reducing sample handling time and contamination risks (Graeber et al., 2025); CRISPR-based diagnostics can enhance sensitivity and specificity (Ghouneimy et al., 2023); AI-assisted analysis can streamline data processing efficiency and improve accuracy (Ogundemuren et al., 2026). Moreover, standardized microbiome classification systems, such as community state types (CSTs), can improve inter-study comparability (Vieira-Baptista et al., 2022). The adoption of these technologies provides fresh momentum for vaginal microbiome studies, but broader dissemination is needed.

### 7.3 Future directions for research on the vaginal microbiome and cervical cancer

Future directions in research on the vaginal microbiome and cervical cancer should emphasize multi-omics integration, large-scale longitudinal studies, precision interventions, and clinical translation. First, multi-omics integration: combining microbiomics, metabolomics, transcriptomics, and proteomics will yield deeper mechanistic insights into how the vaginal microbiome interacts with the host in cervical cancer (Murall et al., 2019). For example, one study integrating microbiomics and metabolomics identified significant correlations between glycerophospholipid metabolites and the abundance of *Prevotella bivia* (Pu et al., 2025). Second, large-scale longitudinal studies: extended follow-up is crucial to delineate causal relationships and the dynamic interplay between the vaginal microbiota and cervical cancer (Murall et al., 2019). The PAPCLEAR cohort study, for instance, plans a 2-year follow-up of 150 women to analyze the dynamic relationship between the microbiota and HPV infection (Murall et al., 2019). Third, precision interventions: personalized probiotic, antibiotic, or VMT regimens based on an individual's microbial signature need development (Medina-Rivero et al., 2026). One study has already shown that probiotic interventions tailored to the individual's microbiota profile can significantly boost HPV clearance (*P* < 0.05) (Dellino et al., 2022).

Clinical translation represents a crucial focal point. This includes developing early cervical cancer screening kits based on the vaginal microbiome (Bik et al., 2019), conducting multi-center clinical trials on probiotic interventions (Abdolalipour et al., 2020), and exploring microbial metabolites as therapeutic targets (Peng et al., 2025). Furthermore, integrating artificial intelligence can refine diagnostic and therapeutic precision (Pawar et al., 2025). Pursuing these research directions will propel the clinical application of vaginal microbiome science in cervical cancer prevention and management, offering new strategies to improve women's health.

## 8 Strengths, limitations, and conclusions

**Strengths:** This review provides a comprehensive and critical synthesis of the current literature on the vaginal microbiome and metabolome in cervical cancer, spanning from basic mechanistic studies to clinical applications. By explicitly distinguishing evidence from meta-analyses and large cohort studies from preliminary or exploratory findings, we offer readers a balanced assessment of the field's progress. The inclusion of epidemiological, diagnostic, and therapeutic perspectives provides a translational framework that may inform both researchers and clinicians.

**Limitations:** As a narrative review, this article has inherent limitations. First, we did not conduct a formal systematic search or meta-analysis, which may introduce selection bias in the studies discussed. Second, the field itself is characterized by heterogeneous study designs, small sample sizes, and variable methodologies (e.g., different 16S rRNA sequencing regions, diverse population groups), which limit the generalisability of findings. Third, most studies are cross-sectional or associative, and causal relationships between vaginal dysbiosis and cervical carcinogenesis remain to be definitively established. Fourth, many of the diagnostic and therapeutic applications discussed are still at experimental or preclinical stages, and their clinical utility requires further validation.

**Conclusions:** Despite these limitations, the accumulating evidence strongly supports the notion that the vaginal microbiome is a significant factor in cervical cancer pathogenesis, influencing HPV persistence and disease progression through immune, inflammatory, and metabolic pathways. Key microbial metabolites-such as short-chain fatty acids, tryptophan catabolites, and lactate-have emerged as potential biomarkers and therapeutic targets. However, translation into clinical practice remains in its early stages. Future research should prioritize large-scale prospective cohorts, standardized methodologies, multi-omics integration, and rigorous clinical trials to validate microbiome-based diagnostics and interventions. With continued effort, vaginal microbiome research holds promise for improving cervical cancer prevention, early detection, and personalized treatment.

## References

[B1] Abdolalipour E. Mahooti M. Salehzadeh A. Torabi A. Mohebbi S. R. Gorji A. . (2020). Evaluation of the antitumor immune responses of probiotic bifidobacterium bifidum in human papillomavirus-induced tumor model. Microb. Pathog. 145:104207. doi: 10.1016/j.micpath.2020.104207 32325236

[B2] Adebamowo S. N. Ma B. Zella D. . (2017). Mycoplasma hominis and mycoplasma genitalium in the vaginal microbiota and persistent high-risk human papillomavirus infection. Front Public Health. 5:140. doi: 10.3389/fpubh.2017.00140 28695118 PMC5483445

[B3] Alhamlan F. S. Albadawi I. A. Al-Qahtani A. A. Awartani K. A. Obeid D. A. Tulbah A. M. (2024). Cervicovaginal and gastrointestinal microbiomes in gynecological cancers and their roles in therapeutic intervention. Front. Microbiol. 15:1489942. doi: 10.3389/fmicb.2024.1489942 39664050 PMC11631898

[B4] Aquino C. I. Nicosia A. Ligori A. Volpicelli A. I. Surico D. (2024). Microbiota status and endometrial cancer: a narrative review about possible correlations in affected versus healthy patients. Science 6:75. doi: 10.3390/sci6040075

[B5] Aslan E. Bechelaghem N. (2018). To “douche” or not to “douche”: hygiene habits may have detrimental effects on vaginal microbiota. J. Obstet. Gynaecol. 38, 678–681. doi: 10.1080/01443615.2017.1395398 29433363

[B6] Audirac-Chalifour A. Torres-Poveda K. Bahena-Román M. Téllez-Sosa J. Martínez-Barnetche J. Cortina-Ceballos B. . (2016). Cervical microbiome and cytokine profile at various stages of cervical cancer: a pilot study. PLoS ONE. 11:e0153274. doi: 10.1371/journal.pone.0153274 27115350 PMC4846060

[B7] Berggrund M. Gustavsson I. Aarnio R. Lindberg J. H. Sanner K. Wikström I. . (2020). Temporal changes in the vaginal microbiota in self-samples and its association with persistent HPV16 infection and CIN2. Virol. J. 17:147. doi: 10.1186/s12985-020-01420-z 33028395 PMC7541248

[B8] Bhattacharya A. Das S. Bhattacharjee M. J. Mukherjee A. K. Khan M. R. (2023). Comparative pangenomic analysis of predominant human vaginal lactobacilli strains towards population-specific adaptation: understanding the role in sustaining a balanced and healthy vaginal microenvironment. BMC Genomics. 24:565. doi: 10.1186/s12864-023-09665-y 37740204 PMC10517566

[B9] Bik E. M. Bird S. W. Bustamante J. P. Leon L. E. Nieto P. A. Addae K. . (2019). A novel sequencing-based vaginal health assay combining self-sampling, HPV detection and genotyping, STI detection, and vaginal microbiome analysis. PLoS ONE. 14:e0215945. doi: 10.1371/journal.pone.0215945 31042762 PMC6493738

[B10] Brooke G. Wendel S. Banerjee A. Wallace N. (2024). Opportunities to advance cervical cancer prevention and care. Tumour Virus Res. 18:200292. doi: 10.1016/j.tvr.2024.200292 39490532 PMC11566706

[B11] Brusselaers N. Shrestha S. van de Wijgert J. Verstraelen H. (2019). Vaginal dysbiosis and the risk of human papillomavirus and cervical cancer: systematic review and meta-analysis. Am. J. Obstet. Gynecol. 221, 9–18.e8. doi: 10.1016/j.ajog.12.011 30550767

[B12] Chalif J. Wang H. Spakowicz D. Quick A. Arthur E. K. O'Malley D. . (2024). The microbiome and gynecologic cancer: cellular mechanisms and clinical applications. Int. J. Gynecol. Cancer. 34, 317–327. doi: 10.1136/ijgc-2023-004894 38088183

[B13] Chao X. Wang L. Wang S. Lang J. Tan X. Fan Q. . (2021). Research of the potential vaginal microbiome biomarkers for high-grade squamous intraepithelial lesion. Front. Med. 8:565001. doi: 10.3389/fmed.2021.565001 34621755 PMC8490638

[B14] Chen L. Wang L. Shen H. Lin H. and Li, D. (2017). Anthelminthic drug niclosamide sensitizes the responsiveness of cervical cancer cells to paclitaxel via oxidative stress-mediated mTOR inhibition. Biochem. Biophys. Res. Commun. 484, 416–421. doi: 10.1016/j.bbrc.2017.01.140 28137584

[B15] Cocomazzi G. Del Pup L. Contu V. Maggio G. Parmegiani L. Ciampaglia W. . (2024). Gynecological cancers and microbiota dynamics: insights into pathogenesis and therapy. Int. J. Mol. Sci.. 25:2237. doi: 10.3390/ijms25042237 38396914 PMC10889201

[B16] Colbert L. E. El Alam M. B. Wang R. Karpinets T. Lo D. Lynn E. J. . (2023). Tumor-resident Lactobacillus iners confer chemoradiation resistance through lactate-induced metabolic rewiring. Cancer Cell. 41, 1945–1962.e11. doi: 10.1016/j.ccell.09.012 37863066 PMC10841640

[B17] da Silva Pinto G. V. Bolpet A. D. N. Martin L. F. Moço N. P. Ramos B. R. A. Silva M. C. . (2023). Factors associated with Trichomonas vaginalis infection in reproductive-aged women attending cervical screening in southeast of Brazil. Braz. J. Infect. Dis. 27:102794. doi: 10.1016/j.bjid.2023.102794 37500061 PMC10412860

[B18] de Carvalho Scharf Santana N. Lima N. A. Desoti V. C. Bidóia D. L. de Souza Bonfim Mendonça P. Ratti B. A. . (2016). Vitamin K3 induces antiproliferative effect in cervical epithelial cells transformed by HPV 16 (SiHa cells) through the increase in reactive oxygen species production. Arch. Gynecol. Obstet. 294, 797–804. doi: 10.1007/s00404-016-4097-7 27091196

[B19] Dellino M. Cascardi E. Laganà A. S. Di Vagno G. Malvasi A. Zaccaro R. . (2022). Lactobacillus crispatus M247 oral administration: is it really an effective strategy in the management of papillomavirus-infected women? Infect. Agents Canc. 17:53. doi: 10.1186/s13027-022-00465-9 PMC9587645 36271433

[B20] Di Pietro M. Filardo S. Porpora M. G. Recine N. Latino M. A. Sessa R. (2018). HPV/Chlamydia trachomatis co-infection: metagenomic analysis of cervical microbiota in asymptomatic women. New Microbiol. 41, 34–41. 29313867

[B21] Donders G. Bellen G. Donders F. Pinget J. Vandevelde I. Michiels T. . (2017). Improvement of abnormal vaginal flora in Ugandan women by self-testing and short use of intravaginal antimicrobials. Eur. J. Clin. Microbiol. Infect. Dis. 36, 731–738. doi: 10.1007/s10096-016-2856-9 27933401

[B22] Ghouneimy A. Mahas A. Marsic T. Aman R. Mahfouz M. (2023). CRISPR-based diagnostics: challenges and potential solutions toward point-of-care applications. ACS Synth. Biol. 12, 1–16. doi: 10.1021/acssynbio.2c00496 36508352 PMC9872163

[B23] Gomes M. Li Z. Olaitan A. Gentry-Maharaj A. (2026). Predictive modeling for cervical cancer: existing AI approaches and the emerging role of vaginal microbiome. Front. Netw. Physiol. 6:1799486. doi: 10.3389/fnetp.2026.1799486 42327708 PMC13278905

[B24] Gong Y. Huang X. Ding Y. Gou F. Tian W. Zi D. (2025). The endometrial microbiota and its influence on the development of endometrial cancer: resolving controversies and elucidating the mechanisms. J. Reprod. Immunol. 172:104745. doi: 10.1016/j.jri.2025.104745 41172772

[B25] Graeber E. Tysha A. Nisar A. Wind D. Mendling W. Finzer P. . (2025). Shallow shotgun metagenomic sequencing of vaginal microbiomes with the oxford nanopore technology enables the reliable determination of vaginal community state types and broad community structures. BMC Microbiol. 25:544. doi: 10.1186/s12866-025-04236-5 40855409 PMC12376446

[B26] Gupta S. Palmer C. Bik E. M. Cardenas J. P. Nuñez H. Kraal L. . (2018). Self-sampling for human papillomavirus testing: increased cervical cancer screening participation and incorporation in international screening programs. Front Public Health. 6:77. doi: 10.3389/fpubh.2018.00077 29686981 PMC5900042

[B27] Ho M. Moon D. Pires-Alves M. Thornton P. D. McFarlin B. L. Wilson B. A. (2021). Recovery of microbial community profile information hidden in chimeric sequence reads. Comput. Struct. Biotechnol. J. 19, 5126–5139. doi: 10.1016/j.csbj.08.050 34589188 PMC8453192

[B28] Huang R. Liu Z. Sun T. Zhu L. (2024). Cervicovaginal microbiome, high-risk HPV infection and cervical cancer: mechanisms and therapeutic potential. Microbiol. Res. 287:127857. doi: 10.1016/j.micres.2024.127857 39121703

[B29] Ilhan Z. E. Łaniewski P. Thomas N. Roe D. J. Chase D. M. Herbst-Kralovetz M. M. (2019). Deciphering the complex interplay between microbiota, HPV, inflammation and cancer through cervicovaginal metabolic profiling. EBioMedicine. 44, 675–690. doi: 10.1016/j.ebiom.2019.04.028 31027917 PMC6604110

[B30] Javadi K. Ferdosi-Shahandashti E. Rajabnia M. Khaledi M. (2024). Vaginal microbiota and gynecological cancers: a complex and evolving relationship. Infect. Agents Canc. 19:27. doi: 10.1186/s13027-024-00590-7 38877504 PMC11179293

[B31] Kang G. U. Jung D. R. Lee Y. H. Jeon S. Y. Han H. S. Chong GO. . (2020). Dynamics of fecal microbiota with and without invasive cervical cancer and its application in early diagnosis. Cancers. 12:3800. doi: 10.3390/cancers12123800 33339445 PMC7766064

[B32] Kang G. U. Jung D. R. Lee Y. H. Jeon S. Y. Han H. S. Chong GO. . (2021). Potential association between vaginal microbiota and cervical carcinogenesis in Korean women: a cohort study. Microorganisms. 9:294. doi: 10.3390/microorganisms9020294 33572693 PMC7912413

[B33] Kao P. Y. Chen J. H. Chen K. H. (2026). The role of HPV and hormone in cervical precancer and cancer: molecular pathophysiology and cell biology of disease and treatment. Oncol. Res. 34:12. doi: 10.32604/or.2026.078219 PMC13220074 42220428

[B34] Karpinets T. V. Solley T. N. Mikkelson M. D. Dorta-Estremera S. Nookala S. S. Medrano A. Y. D. . (2020). Effect of antibiotics on gut and vaginal microbiomes associated with cervical cancer development in mice. Cancer Prev Res. 13, 997–1006. doi: 10.1158/1940-6207.CAPR-20-0103 PMC10583130 32917644

[B35] Kero K. Rautava J. Syrjänen K. Grenman S. Syrjänen S. (2017). Association of asymptomatic bacterial vaginosis with persistence of female genital human papillomavirus infection. Eur. J. Clin. Microbiol. Infect. Dis. 36, 2215–2219. doi: 10.1007/s10096-017-3048-y 28681204

[B36] Łaniewski P. Barnes D. Goulder A. Cui H. Roe D. J. Chase DM. . (2018). Linking cervicovaginal immune signatures, HPV and microbiota composition in cervical carcinogenesis in non-hispanic and hispanic women. Sci. Rep. 8:7593. doi: 10.1038/s41598-018-25879-7 29765068 PMC5954126

[B37] Liao B. Chen L. Ruan J. Wang R. Hu B. Long R. . (2026). Microbiome and gartynecologic cancer. Cancer Lett. 636:217940. doi: 10.1016/j.canlet.2025.217940 40692066

[B38] Lin G. Lin YC. Wu RC. Yang LY. Lu HY. Tsai SY. . (2018). Developing and validating a multivariable prediction model to improve the diagnostic accuracy in determination of cervical versus endometrial origin of uterine adenocarcinomas: a prospective MR study combining diffusion-weighted imaging and spectroscopy. J. Magn. Reson. Imaging. 47, 1654–1666. doi: 10.1002/jmri.25899 29178414

[B39] Lin L. Lin X. Lin L. Feng Q. Kitamori T. Lin JM. . (2017). Integrated microfluidic platform with multiple functions to probe tumor-endothelial cell interaction. Anal. Chem. 89, 10037–10044. doi: 10.1021/acs.analchem.7b02593 28820578

[B40] Linn Y. H. Thu K. K. Win N. H. H. (2019). Effect of probiotics for the prevention of acute radiation-induced diarrhoea among cervical cancer patients: a randomized double-blind placebo-controlled study. Probiotics Antimicrob. Proteins. 11, 638–647. doi: 10.1007/s12602-018-9408-9 29550911

[B41] Liu J. Mosavati B. Oleinikov A. V. Du E. (2019). Biosensors for detection of human placental pathologies: a review of emerging technologies and current trends. Transl. Res. 213, 23–49. doi: 10.1016/j.trsl.2019.05.002 31170377 PMC6783355

[B42] Ma Y. Wan L. Li R. Chen X. Wang H. (2024). Impact of postsurgical vaginal microbiome on high-risk HPV infection and recurrence risk in patients with cervical cancer and intraepithelial neoplasia: a retrospective study. Gynecol Oncol Rep. 55:101506. doi: 10.1016/j.gore.2024.101506 39308899 PMC11416653

[B43] Medina-Rivero A. S. Ratkovich-Gonzalez S. Ruiz-Briseño M. D. R. Rosas-González V. C. (2026). The role of cervicovaginal microbiota and probiotics in modulating HPV persistence and preventing cervical cancer. ACS Infect. Dis. 12, 878–896. doi: 10.1021/acsinfecdis.5c00385 41160423

[B44] Meng L. Xu Y. Lin Z. Shen Y. Zhang Y. Liu S. (2026). Microbiota dynamics in HPV-Infected individuals: implications for cervical neoplasia development. Discov Oncol. 17:451. doi: 10.1007/s12672-026-04641-w 41686340 PMC13004766

[B45] Menga A. Palmieri E. M. Cianciulli A. Infantino V. Mazzone M. Scilimati A. . (2017). SLC25A26 overexpression impairs cell function via mtDNA hypermethylation and rewiring of methyl metabolism. FEBS J. 284, 967–984. doi: 10.1111/febs.14028 28118529

[B46] Menon S. Broeck DV. Rossi R. Ogbe E. Harmon S. Mabeya H. (2016). Associations between vaginal infections and potential high-risk and high-risk human papillomavirus genotypes in female sex workers in Western Kenya. Clin. Ther. 38, 2567–2577. doi: 10.1016/j.clinthera.2016.10.005 27836494

[B47] Mitra A. MacIntyre D. A. Marchesi J. R. Lee Y. S. Bennett P. R. Kyrgiou M. (2016). The vaginal microbiota, human papillomavirus infection and cervical intraepithelial neoplasia: what do we know and where are we going next? Microbiome. 4:58. doi: 10.1186/s40168-016-0203-0 27802830 PMC5088670

[B48] Mocciaro V. Scollo P. Stefano A. Gieri S. Russo G. Scibilia G. . (2016). Correlation between histological grade and positron emission tomography parameters in cervical carcinoma. Oncol. Lett. 12, 1408–1414. doi: 10.3892/ol.2016.4771 27446445 PMC4950245

[B49] Moghaddam N. A. Mohabbat A. Jigheh M. P. Mahboubi A. Ravanlo Z. Z. Shanehbandi D. . (2026). The interplay between HPV vaginal microbiota and host immunity in cervical carcinogenesis. Discov. Oncol. 17:5169. doi: 10.1007/s12672-026-05169-9 PMC13433681 42223784

[B50] Molina M. A. Steenbergen R. D. M. Pumpe A. Kenyon A. N. Melchers W. J. G. (2024). HPV integration and cervical cancer: a failed evolutionary viral trait. Trends Mol. Med. 30, 890–902. doi: 10.1016/j.molmed.2024.05.009 38853085

[B51] Montecillo J. A. V. Yoo H. J. Lee Y. Y. Park C. M. Cho A. Lee H. . (2026). Streptococcus vaginalis affects cellular dynamics of cervical cancer cells via oxidative stress-induced activation of endoplasmic reticulum unfolded protein response. Microbiol. Res. 305:128433. doi: 10.1016/j.micres.2025.128433 41455276

[B52] Murall C. L. Rahmoun M. Selinger C. Baldellou M. Bernat C. Bonneau M. . (2019). Natural history, dynamics, and ecology of human papillomaviruses in genital infections of young women: protocol of the PAPCLEAR cohort study. BMJ Open. 9:e025129. doi: 10.1136/bmjopen-2018-025129 31189673 PMC6576111

[B53] Negi D. Singh A. Joshi N. Mishra N. (2020). Cisplatin and probiotic biomass loaded pessaries for the management of cervical cancer. Anticanc. Agents Med. Chem. 20, 589–598. doi: 10.2174/1871520619666191211110640 31823703

[B54] Nicolò S. Tanturli M. Mattiuz G. Antonelli A. Baccani I. Bonaiuto C. . (2021). Vaginal lactobacilli and vaginal dysbiosis-associated bacteria differently affect cervical epithelial and immune homeostasis and anti-viral defenses. Int. J. Mol. Sci. 22:6487. doi: 10.3390/ijms22126487 34204294 PMC8234132

[B55] Ogundemuren D. A. Agrahari V. Wong A. P. Herrera C. Ilomuanya M. O. Doncel G. F. (2026). Artificial intelligence and machine learning in smart vaginal formulation development. Adv. Drug Deliv. Rev. 234:115882. doi: 10.1016/j.addr.2026.115882 42055249

[B56] Onywera H. Williamson A. L. Mbulawa Z. Z. A. Coetzee D. Meiring T. L. (2019). Factors associated with the composition and diversity of the cervical microbiota of reproductive-age Black South African women: a retrospective cross-sectional study. PeerJ. 7:e7488. doi: 10.7717/peerj.7488 31435492 PMC6698374

[B57] Pawar M. Pingale S. Kadam K. Jadhav A. Kaul-Ghanekar R. (2025). Artificial intelligence-driven screening, early diagnosis, and treatment strategies for cervical cancer: an overview. Infect. Agents Canc. 21:1. doi: 10.1186/s13027-025-00716-5 41316410 PMC12763967

[B58] Peng K. Chen X. Zhang Y. Liu L. Huang Q. Du S. . (2025). Lactobacillus crispatus-derived nCEV vesicles promote cutaneous wound healing and inhibit HPV16 infection. mSystems. 10:e0068325. doi: 10.1128/msystems.00683-25 40827924 PMC12455947

[B59] Pourmollaei S. Barzegari A. Farshbaf-Khalili A. Nouri M. Fattahi A. Shahnazi M. . (2020). Anticancer effect of bacteria on cervical cancer: molecular aspects and therapeutic implications. Life Sci. 246:117413. doi: 10.1016/j.lfs.2020.117413 32035929

[B60] Pu X. Wang X. Wang J. Gu Z. Zhu H. Li C. (2025). Microbial and metabolic profiles associated with HPV infection and cervical intraepithelial neoplasia: a multi-omics study. Microbiol Spectr. 13:e0019225. doi: 10.1128/spectrum.00192-25 40304477 PMC12131762

[B61] Qiu G. Yu Y. Wang Y. Wang X. (2019). The significance of probiotics in preventing radiotherapy-induced diarrhea in patients with cervical cancer: a systematic review and meta-analysis. Int. J. Surg. 65, 61–69. doi: 10.1016/j.ijsu.2019.03.015 30928672

[B62] Ricci C. Scaldaferri F. Colombo F. Armuzzi A. Lopetuso L. R. Leone S. . (2020). Prevalence of cervical HPV and attitude towards cervical screening in IBD patients under immunomodulatory treatment: a multidisciplinary management experience. Eur. Rev. Med. Pharmacol. Sci. 24, 564–570. doi: 10.26355/eurrev_202001_20032 32016957

[B63] Rodríguez-Esquivel M. Rosales J. Castro R. Apresa-García T. Garay Ó. Romero-Morelos P. . (2018). Volatolome of the female genitourinary area: toward the metabolome of cervical cancer. Arch. Med. Res. 49, 27–35. doi: 10.1016/j.arcmed.2018.04.004 29681412

[B64] Sasidharan B. K. Ramadass B. Viswanathan P. N. Samuel P. Gowri M. Pugazhendhi S. . (2019). A phase 2 randomized controlled trial of oral resistant starch supplements in the prevention of acute radiation proctitis in patients treated for cervical cancer. J. Cancer Res. Ther. 15. doi: 10.4103/jcrt.JCRT_152_19 31898677

[B65] Sharifian K. Shoja Z. Jalilvand S. (2023). The interplay between human papillomavirus and vaginal microbiota in cervical cancer development. Virol. J. 20:73. doi: 10.1186/s12985-023-02037-8 37076931 PMC10114331

[B66] Sun Z. Qi D. Liu L. Ai W. Han B. Wang S. . (2025). Role of the ALK gene and PI3K/Akt/NF-κB signaling pathway in cervical cancer precancerous lesions. Front. Oncol. 15:1619703. doi: 10.3389/fonc.2025.1619703 40978038 PMC12443549

[B67] Tossas K. Y. Zhu B. Perera R. A. Serrano M. G. Sullivan S. Sayeed S. . (2023). Does the vaginal microbiome operate differently by race to influence risk of precervical cancer? J. Womens Health. 32, 553–560. doi: 10.1089/jwh.2022.0309 36897755 PMC10171949

[B68] Tsementzi D. Meador R. Eng T. Shelton J. Scott I. Konstantinidis KT. . (2025). Associations among HPV persistence, the vaginal microbiome, and cervical cancer recurrence. J. Transl. Med. 23:858. doi: 10.1186/s12967-025-06811-w 40750891 PMC12317609

[B69] Usyk M. Zolnik C. P. Castle P. E. Porras C. Herrero R. Gradissimo A. . (2020). Cervicovaginal microbiome and natural history of HPV in a longitudinal study. PLoS Pathog. 16:e1008376. doi: 10.1371/journal.ppat.1008376 32214382 PMC7098574

[B70] van de Wijgert J. H. H. M. Gill A. C. Chikandiwa A. Verwijs M. C. Kelly H. A. Omar T. . (2020). Human papillomavirus infection and cervical dysplasia in HIV-positive women: potential role of the vaginal microbiota. AIDS. 34, 115–125. doi: 10.1097/QAD.0000000000002381 31567164

[B71] Vieira-Baptista P. De Seta F. Verstraelen H. Ventolini G. Lonnee-Hoffmann R. Lev-Sagie A. (2022). The vaginal microbiome: v. therapeutic modalities of vaginal microbiome engineering and research challenges. J. Low Genit. Tract. Dis. 26, 99–104. doi: 10.1097/LGT.0000000000000647 34928260 PMC8719494

[B72] Walker H. Burrell M. Flatley J. Powers H. (2017). A metabolite profiling method for diagnosis of precancerous cervical lesions and HPV persistence. Bioanalysis. 9, 601–608. doi: 10.4155/bio-2017-0012 28508693

[B73] Wang H. Ma Y. Li R. Chen X. Wan L. Zhao W. (2019). Associations of cervicovaginal lactobacilli with high-risk human papillomavirus infection, cervical intraepithelial neoplasia, and cancer: a systematic review and meta-analysis. J. Infect. Dis. 220, 1243–1254. doi: 10.1093/infdis/jiz325 31242505

[B74] Wang S. Xie J. Li J. Liu F. Wu X. Wang Z. (2016). Cisplatin suppresses the growth and proliferation of breast and cervical cancer cell lines by inhibiting integrin β5-mediated glycolysis. Am. J. Cancer Res. 6, 1108–1117. 27294003 PMC4889724

[B75] Wei B. Chen Y. Lu T. Cao W. Tang Z. Yang H. (2022). Correlation between vaginal microbiota and different progression stages of cervical cancer. Genet. Mol. Biol. 45:e20200450. doi: 10.1590/1678-4685-gmb-2020-0450 35320337 PMC8967114

[B76] Wierzbińska W. Kuzmycz O. Kowalczyk A. Staczek P. (2025). The role of dysbiosis in shaping host immunity in endometrial cancer development. Front. Immunol. 16:1627285. doi: 10.3389/fimmu.2025.1627285 41050662 PMC12491265

[B77] Xie Y. Feng Y. Li W. Zhan F. Huang G. Hu H. . (2020). Revealing the disturbed vaginal micobiota caused by cervical cancer using high-throughput sequencing technology. Front. Cell. Infect. Microbiol. 10:538336. doi: 10.3389/fcimb.2020.538336 33365275 PMC7750457

[B78] Xin M. Qiao Z. Li J. Liu J. Song S. Zhao X. . (2016). miR-22 inhibits tumor growth and metastasis by targeting ATP citrate lyase: evidence in osteosarcoma, prostate cancer, cervical cancer and lung cancer. Oncotarget. 7, 44252–44265. doi: 10.18632/oncotarget.10020 27317765 PMC5190093

[B79] Xu H. Liu L. Xu F. Liu M. Song Y. Chen J. . (2022). Microbiome-metabolome analysis reveals cervical lesion alterations. Acta Biochim. Biophys. Sin. 54, 1552–1560. doi: 10.3724/abbs.2022149 36269135 PMC9828295

[B80] Yang K. Xia B. Wang W. Cheng J. Yin M. Xie H. . (2017). A comprehensive analysis of metabolomics and transcriptomics in cervical cancer. Sci. Rep. 7:43353. doi: 10.1038/srep43353 28225065 PMC5320559

[B81] Yang M. Li L. Jiang C. Qin X. Zhou M. Mao X. . (2020). Co-infection with trichomonas vaginalis increases the risk of cervical intraepithelial neoplasia grade 2-3 among HPV16 positive female: a large population-based study. BMC Infect. Dis. 20:642. doi: 10.1186/s12879-020-05349-0 32873233 PMC7466445

[B82] Yockey L. J. Hussain F. A. Bergerat A. Reissis A. Worrall D. Xu J. . (2022). Screening and characterization of vaginal fluid donations for vaginal microbiota transplantation. Sci. Rep. 12:17948. doi: 10.1038/s41598-022-22873-y 36289360 PMC9606370

[B83] Yoysungnoen B. Bhattarakosol P. Changtam C. Patumraj S. (2016). Effects of tetrahydrocurcumin on tumor growth and cellular signaling in cervical cancer xenografts in nude mice. Biomed Res. Int. 2016:1781208. doi: 10.1155/2016/1781208 26881213 PMC4736311

[B84] Zhang R. Su J. Xue S. L. Yang H. Ju L. L. Ji Y. . (2016). HPV E6/p53 mediated down-regulation of miR-34a inhibits Warburg effect through targeting LDHA in cervical cancer. Am. J. Cancer Res. 6, 312–320. 27186405 PMC4859662

[B85] Zhang Z. Ma Q. Zhang L. Ma L. Wang D. Yang Y. . (2024). Human papillomavirus and cervical cancer in the microbial world: exploring the vaginal microecology. Front. Cell. Infect. Microbiol. 14:1325500. doi: 10.3389/fcimb.2024.1325500 38333037 PMC10850380

[B86] Zhang Z. Zhang D. Xiao B. B. Zhang R. Bai H. H. Dong H. Y. . (2018). [Primary study on the relationship between high-risk HPV infection and vaginal cervical microbiota]. Zhonghua Fu Chan Ke Za Zhi. 53, 471–480. doi: 10.3760/cma.j.issn.0529-567x.2018.07.006 30078257

[B87] Zhao Y. Wang X. Li L. Li C. (2016). Doxycycline inhibits proliferation and induces apoptosis of both human papillomavirus positive and negative cervical cancer cell lines. Can. J. Physiol. Pharmacol. 94, 526–533. doi: 10.1139/cjpp-2015-0481 26913972

[B88] Zhen Q. Xu Y. Xu Y. Liu X. Zhang Y. Ye D. . (2025). Erucic acid, derived by *Lactobacillus Crispatus*, induces ferroptosis in cervical cancer organoids through the ppar-δ signaling pathway. Adv. Sci. 12:e12599. doi: 10.1002/advs.202512599 41082335 PMC12752652

